# Prolactin and Hyperprolactinaemia in Endometriosis-Related Infertility: Are There Clinically Significant Connections?

**DOI:** 10.3390/jcm13195868

**Published:** 2024-10-01

**Authors:** Ranko Kutlesic, Marija Kutlesic, Jelena Milosevic-Stevanovic, Predrag Vukomanovic, Milan Stefanovic, Danka Mostic-Stanisic

**Affiliations:** 1 Department of Gynaecology and Obstetrics, Faculty of Medicine, University of Nis, 18000 Nis, Serbia; 2 Clinic of Gynaecology and Obstetrics, University Clinical Centre Nis, 18000 Nis, Serbia; 3 Institute of Gynaecology and Obstetrics Belgrade, University Clinical Centre of Serbia, 11000 Belgrade, Serbia; 4 Faculty of Medicine, University of Belgrade, 11000 Belgrade, Serbia

**Keywords:** endometriosis, prolactin, hyperprolactinaemia, infertility, female

## Abstract

Endometriosis and hyperprolactinaemia are conditions that might lead to infertility as a consequence. The aim of this article was to present the current knowledge about possible relationships between prolactin/hyperprolactinaemia and endometriosis-related infertility. Experimental studies on local prolactin acting as cytokine and relationship of prolactin and endometriotic tissue, as well as clinical studies on hyperprolactinaemia and endometriosis-related infertility suggest the possible role of prolactin in endometriosis-related infertility, but final proof is still missing and the exact pathogenesis of infertility in such cases is still under investigation. Novel strategies in the treatment of endometriosis-related infertility, based on its connection with prolactin such as the use of prolactin receptor antibodies and prolactin receptor antagonists, are under investigation, but adequate clinical studies have yet to be undertaken.

## 1. Introduction

Endometriosis and hyperprolactinaemia are conditions that might lead to infertility as a consequence.

Endometriosis, the presence of endometrial-like tissue outside the uterus [1], is a common condition in women of reproductive age, among which the prevalence is estimated to be 5–10%, and is frequently diagnosed in women with infertility, especially in association with a higher risk of subsequent infertility in women aged < 35 years [1].

Hyperprolactinaemia is an excess of prolactin levels in blood serum, which can be associated with menstrual cycle abnormalities that can progress sequentially as the level of prolactin increases: from an inadequate luteal phase with almost regular cycles to intermittent anovulation with oligomenorrhoea to total anovulation and amenorrhoea. The consequences are subfertility and infertility: hyperprolactinaemia can be identified in 5% of female patients presenting with infertility [2]. The impact of prolactin and hyperprolactinaemia on endometriosis-induced infertility has been studied for more than 40 years, but a clear relationship has not been established, even though the association of galactorrhoea and endometriosis was reported for the first time more than 40 years ago [3].

The results of studies on the possible connection and involvement of prolactin in the development of endometriosis are contradictory. Some studies have reported a positive correlation of the basal and stimulated prolactin serum levels with endometriosis and stages of the disease, but there have also been studies that failed to prove such connections (Table 1). The authors of the recent review, who focused on the connection of prolactin and uterine pathology, stated that prolactin is “clearly implicated in endometriosis-associated infertility” [4].

According to the recent research on the action of local prolactin as an inflammatory, angiogenic, and mitogenic factor [19], it is more probable that local, and not systemic prolactin, is involved in the pathogenesis of endometriosis and associated infertility. Still, systemic hyperprolactinaemia is known to disturb follicular development, ovulation, and function of the corpus luteum in humans, so it could contribute to the development of infertility in endometriosis patients without tubal occlusion.

The aim of this article was to present the current knowledge about possible relationships between hyperprolactinaemia and endometriosis-induced infertility.

## 2. Methods

This study is a narrative review, where clinical and experimental studies on prolactin, hyperprolactinaemia, and endometriosis are presented. The primary aim was to present clinical studies on infertile patients with hyperprolactinaemia and endometriosis, particularly focused on the connection between prolactin levels and endometriosis and the effects of the treatment for hyperpolactinaemia on endometriosis and associated infertility. The secondary aim was to present the results of experimental studies that focused on the connection of systemic/local prolactin action and the development of ectopic endometrium as well as a possible treatment options.

The available literature on prolactin, hyperprolactinaemia, and endometriosis was analysed. A literature search was conducted using MEDLINE via PubMed and Google Scholar databases covering the period from 1 January 1976 to 1 July 2024 including articles in any language. Two reviewers (RK and MK) independently searched through the literature using the terms: “Hyperprolactinemia” [Mesh]) OR “Prolactin” [Mesh]) AND “Endometriosis” [Mesh].

All clinical studies that did not establish a diagnosis of hyperprolactinaemia (prolactin serum levels > 530 mIU/L or >25 ng/mL) according to the World Health Organisation Standard 84/500 and previous recommendations [20] were excluded. All clinical studies that did not confirm a diagnosis of endometriosis by laparoscopy were excluded. Abstracts were also excluded.

## 3. Pituitary and Extrapituitary Prolactin Production and Action

PRL may have pituitary and extrapituitary origins. The main sources of prolactin in humans are anterior pituitary lactotrophs. The synthesis and secretion of pituitary PRL are regulated by inhibiting and releasing factors.

Inhibiting factors include dopamine, gamma-aminobutyric acid (GABA), and somatostatin. Hypothalamic dopamine is considered to be the main prolactin inhibiting factor in humans, exerting its action through D2 receptors located on the cell membranes of lactotrophs. Such dopamine action results in the downregulation of PRL gene expression, reduced PRL secretion, and decreased lactotroph proliferation [19].

Different molecules release pituitary prolactin: thyrotropin-releasing hormone (being the most important clinically), oxytocin, galanin, salsolinol, vasoactive intestinal polypeptide and others, but the “PRL-releasing factor” has not been identified, and none of the mentioned factors have been proven to meet the criteria to be considered as hypothalamic hypophysiotrophic factors [21]. Prolactin mediates its effects by the prolactin receptor, which belongs to the class 1 cytokine receptor superfamily, and these receptors have three different isoforms: the short, the long, and the intermediate form, which differ by the length of their cytoplasmic domains. Prolactin receptors are widely distributed in the body, which is in agreement with the actions of prolactin in vertebrates on endocrinology, reproduction, water and salt balance, growth and development, metabolism, brain and behaviour, and immune regulation [22]. Prolactin modulates the reproductive function at the central level, acting on a specific population of hypothalamic arcuate nuclei neurons that express the Kiss1 gene, encoding neuropeptides kisspeptins, which are potent stimulators of GnRH neurons. Hyperprolactinaemia induces the suppression of Kiss1 mRNA expression and kisspeptin secretion, leading to a lower stimulation of GnRH neurons, the inhibition of GnRH secretion, diminished GnRH receptor response, and a decline in luteinising hormone pulse frequency and amplitude [23]. In primates, prolactin does not play a significant role in the regulation of corpus luteum, although there is a possibility that prolactin could be part of a ‘’luteotrophic complex’’ together with luteinising and follicle stimulating hormone [24].

**Scheme 1:**
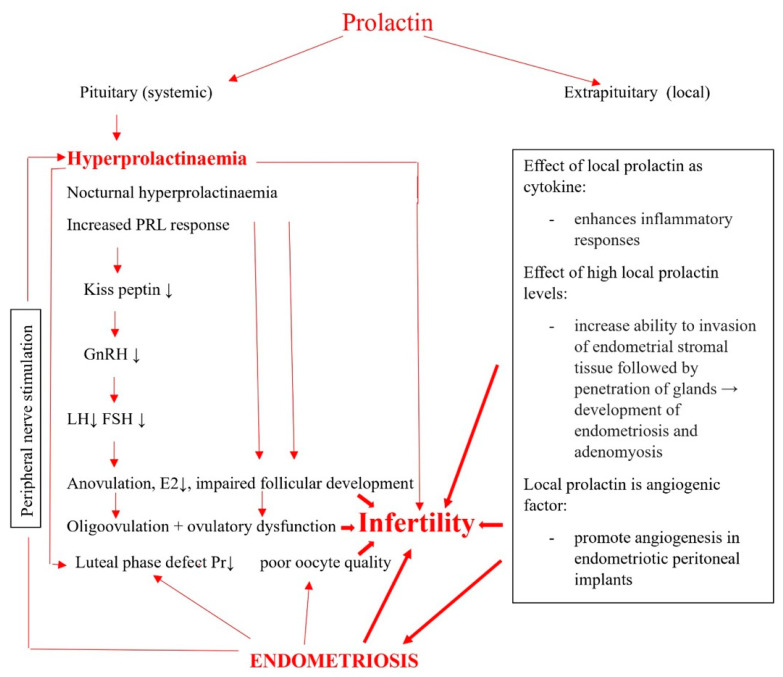
Connection between prolactin and endometriosis-related infertility: impact of increased prolactin serum levels and disturbed regulation of local prolactin on endometriosis.

On the other hand, hyperprolactinaemia inhibits basal and gonadotropin stimulated steroidogenesis in the human ovary, and this may be one cause of the hypogonadism and infertility associated with hyperprolactinemia [25] (Scheme 1).

Hyperprolactinaemia also inhibits the development of corpus luteum and granulose cell luteinisation, and hyperprolactinaemia associated luteal phase defects may not only be the consequence of prolactin suppression of the reproductive function on the central level, but may be the consequence of an inappropriate elevated prolactin level in the follicular microenvironment [26]. The consequences are impaired follicular development, dysregulation in steroidogenesis, ovulatory dysfunction from anovulation to oligovulation, and luteal phase defects. All of the above-mentioned lead to subfertility and infertility (Scheme 1). Extrapituitary prolactin is structurally identical to pituitary PRL and is produced in the ovaries, uterus (both in myometrium and endometrium), breast, prostate, lymphocytes, haematopoietic cells, adipose tissue, skin, thymus, lymphatic system, endothelium, and the brain. The action is still under investigation, which is most probably autocrine/paracrine in nature, but has been well-established that its regulation is site-specific and quite different from the pituitary PRL [27].

The secretory endometrium synthetises prolactin by the direct action of progesterone, and the synthesis of endometrial prolactin is directly correlated to the progesterone induced differentiation of stromal cells in both normoprolactinaemic and hyperprolactinaemic women [28], which leads to the conclusion that the regulation of endometrial prolactin is autocrine and different from the regulation of pituitary PRL secretion [29].

The human ovary is also a site of local prolactin production, with significantly increased concentrations of prolactin in premenopausal ovarian tissue in comparison to postmenopausal, suggesting the role of prolactin as not only a hormone, but also as an autocrine or paracrine regulatory factor providing minimal luteal support [30]. The physiological levels of local prolactin in most rodents potentiate the steroidogenic effects of luteinising hormone on granulosa-luteal cells and inhibit the 20-hydroxysteroid dehydrogenase enzyme, which inactivates progesterone [19].

## 4. Peripheral Prolactin and Reproductive Failure

The impact of excessive pituitary prolactin on reproductive failure is well-known. Extrapituitary or peripheral prolactin has similar chemical, biological, and immunological properties as pituitary prolactin, suggesting the possible role of peripheral prolactin in the development of subfertility and infertility, but the exact mechanisms are not fully understood [4].

It has been reported that the total lack of prolactin in the reproductive microenvironment is devastating for fertility. An experimental study conducted by Ormandy et al. [31] supports the role of peripheral prolactin in reproductive function. Female mice carrying a germ-line null mutation of the prolactin receptor gene presented multiple reproductive anomalies including not only irregular cycles, but fewer follicles in their ovaries, reduced ovulation and fertilisation, defective preimplantation development of embryos as well as sterility, suggesting that prolactin has an important influence on oocyte maturation and atresia. Defective preimplantation development of their embryos is caused by a deficient environment in the oviduct. Moreover, the small number of embryos that progress to the blastocyst stage are released into the uterus refractory to implantation, the consequence of which is infertility.

The role of endometrial prolactin has been investigated, especially in endometrial receptivity. It has been suggested that the lack of endometrial prolactin expression might be involved in reproductive failure [32]. This hypothesis is supported by the study on endometrial samples collected by hysteroscopy in the late luteal phase in patients with unexplained infertility and repeated miscarriages, which reported the lack of endometrial prolactin expression during the “implantation window’’ [33].

It has also been reported that patients with recurrent pregnancy loss associated with antiphospholipid syndrome have a significantly lower expression of decidual prolactin, suggesting impaired endometrial differentiation before conception [34].

On the other hand, it seems that the dysregulation of local prolactin receptor signalling or higher levels of local prolactin could also impair reproduction, acting as a proinflammatory cytokine and angiogenic factor [19], thus contributing to the development of endometriosis or as an immunomodulating factor that participates in activating a local inflammatory environment favourable for the persistence of endometriosis. The results of studies on this issue will be presented in the next two sections.

## 5. The Impact of Prolactin on Endometriosis—Physiological Bases

The endometrium has prolactin receptors and is capable of producing prolactin, so it was hypothesised that this was also the case with endometriotic implants, but the results of the studies on this issue have shown the opposite.

The expression of prolactin receptors in the normal endometrium is weak during the proliferative phase and increases during the secretory phase after the 22nd day of the cycle [35]. In a recent study, Otto et al. compared the in vivo effects of the prolactin receptor antibodies used in an endometriosis interna (or adenomyosis) model in SHN mice [36]. SHN mice are a strain of mice with high mammary tumour potentials, established from basal stock of the Swiss albino and registered internationally [37], and these mice develop endometriosis interna (adenomyosis) spontaneously with increasing age. The authors previously generated the hypothesis that not only systemic hyperprolactinaemia, but also enhanced local prolactin-mediated signalling in endometriotic lesions, might contribute to the development of endometriosis [38], and concluded that prolactin receptor blockade completely inhibited endometriosis in this mouse model to the same extent as the anti-oestrogen faslodex or the GnRH antagonist cetrorelix. 

The results of other experimental studies also support the role of prolactin in the aetiology and pathogenesis of endometriosis. Studies on mice models have shown the induction of adenomyosis by intrauterine pituitary isografts [39,40] and after treatment with dopamine antagonists [41], suggesting the role of prolactin in the development of adenomyosis. 

It has been proposed that hyperprolactinaemic state, which is the consequence of pituitary grafting, can lead to the invasion of endometrial stromal tissue followed by the penetration of glands through invaded area. The uterine horn, contralateral to the implanted pituitary graft, also develops adenomyosis as well as pituitary transplantation under the renal capsule [42], suggesting that generalised hyperprolactinaemia is involved in the development of adenomyosis. A possible explanation for the formation of adenomyosis in these mice is the action of elevated prolactin levels to increase the ability of endometrial stromal cells to invade the myometrium through degradation of the extracellular matrix with matrix metalloproteinases. Thus, elevated prolactin levels may lead to the increased ability of endometrial cells to invade the myometrium through the degradation of the extracellular matrix with MMPs [43] (Scheme 1).

The results of some other studies on prolactin’s role in the development of endometriosis do not support the hypothesis that prolactin is involved in the formation of endometriosis. One of the first studies on prolactin receptors in endometrium and endometriotic tissue reported that prolactin receptors were expressed in the human endometrium, but that the expression of prolactin receptors was lower in human endometriotic tissue at least during the mid-late proliferative phase of the menstrual cycle in women with laparoscopically-proven endometriosis. According to the results of this study, a prolactin receptor transcript was present in 74% of the normal endometrium samples and absent in 85% of the endometriotic samples, using each woman as her own control. The authors of the study concluded that the absence of prolactin receptors in endometriotic tissue could not be correlated with the serum prolactin levels, suggesting the existence of a different regulation of prolactin receptor expression in endometriotic tissue compared with a normal endometrium [44]. This finding is consistent with the results of a previously mentioned study that the expression of prolactin receptors in the endometrium was weak during proliferative phase [35] On the other hand, the oestrogen and progesterone receptors were reported to be significantly lower or absent in an endometriotic endometrium compared to a normal endometrium [45].

It was also demonstrated that the expression of prolactin receptors in normal human endometrium was stimulated upon progesterone-induced PRL gene expression in endometrial stromal cells, supporting the hypothesis that PRL may have an autocrine effect in the endometrium and decidua [46].

The positive impact of oestradiol on increased cell proliferation in endometriosis and adenomyosis is well-known. It has been reported that the upregulation of prolactin synthesis in adenomyosis takes place in the same manner as in the normal endometrium [47].

## 6. Prolactin as an Immunomodulating Factor in Endometriosis-Related Infertility

Prolactin is an upregulator of immune processes, and hyperprolactinaemia is often present in autoimmune diseases. Acting as a cytokine, prolactin is one of the mediators of the communication between neuroendocrine and immune systems and participates in many immunomodulatory activities, affects the differentiation and maturation of both B and T lymphocytes, and enhances inflammatory responses and the production of immunoglobulins [48].

Prolactin also increases the secretion of cytokines, IL-1β, IL-6 (stimulators), TNF-α, and interferon-γ from various cells [49,50]. It has also been reported that cytokines IL-1β and IL-6 can stimulate prolactin secretion [51].

Hyperprolactinemia has been described in many autoimmune diseases, as in systemic lupus erythematosus, rheumatoid arthritis and psoriatic arthritis as well as in organ specific diseases [52,53,54].

On the other hand, endometriosis is a chronic, inflammatory, and hormone-dependent disease, and the immune system (systemically and locally in endometrium, pelvic endometriotic lesions, and peritoneal fluid) is believed to play a central role in its aetiology, pathophysiology, and the associated morbidities of pain, infertility, and poor pregnancy outcomes [55]. In fact, the association of hormonal and immune factors activates the local inflammatory microenvironment, which enables the persistence of endometriotic implants [56]. Taken together, these data are highly suggestive of the possible connection between local prolactin action and the development of endometriosis-induced infertility. Further clinical studies involving patients with endometriosis-induced infertility and hyperprolactinaemia focused on immune response would provide the answers to this connection and possible treatment options.

## 7. Clinical Studies on Relationship between Prolactin/Hyperprolactinaemia, Endometriosis, and Endometriosis-Related Infertility

The impact of prolactin and hyperprolactinaemia on endometriosis and endometriosis-related infertility has been studied for more than 40 years, but a clear relationship has not been established. One of the first clinical reports on this issue described ‘’galactorrhoea-endometriosis syndrome” in nine patients with endometriosis: eight had galactorrhoea either at diagnosis or after treatment with danazol, moreover, in seven of them, the prolactin serum concentrations were normal. The authors concluded that “galactorrhoea in women with regular menstrual periods should suggest a possible diagnosis of underlying endometriosis, especially if there is a history of dysmenorrhea” [3]. A possible explanation for the normal prolactin serum levels in the seven reported patients with normal fasting prolactin values and described “galactorrhoea-endometriosis syndrome” could be the possibility of nocturnal hyperprolactinaemia: in such patients, the nocturnal prolactin peak is more exaggerated than the physiological prolactin peak during nocturnal sleep. Other authors have suggested that nocturnal hyperprolactinaemia is a cause of luteal insufficiency and galactorrhoea [57] as well as infertility in patients with normoprolactinemic galactorrhoea [58].

Since this early report, studies have sought to identify the relationship between prolactin serum levels and endometriosis as well as the validity of serum prolactin levels in diagnosing endometriosis and whether there are differences in the prolactin serum levels regarding stage of the disease.

One clinically important question is also the effect of the endometriosis treatment on the prolactin serum levels as well as the question of whether the medical treatment of hyperprolactinaemia could be useful in improving endometriosis and fertility in endometriosis patients.

Most of the studies that measured the basal prolactin serum levels in patients with and without endometriosis reported higher basal prolactin serum levels in patients with endometriosis compared to healthy controls [7,12,13,15,18] (Table 1). One of the first studies on this issue reported 2-fold greater baseline prolactin concentrations in the infertile patients with endometriosis compared to the normal fertile controls, but the difference was not statistically significant. Following TRH administration, a significant increase in prolactin serum concentration was observed in patients with endometriosis, suggesting that some patients with endometriosis exhibit a greater capacity for prolactin secretion than normal women. Interestingly, this hypersecretory state was selective for prolactin, and no significant differences between the baseline and TRH-stimulated thyroid-stimulating hormone concentrations or total serum thyroxine concentrations were observed [5].

In another early study, the basal PRL serum levels were measured in 16 normal women and 60 women with endometriosis (37 with infertility). There was no difference between the normal women and patients with mild endometriosis, but patients with moderate endometriosis had increased basal PRL serum levels compared with the normal controls and 47% of them had hyperprolactinaemia. There were no significant differences between patients with moderate and severe endometriosis. Among the patients with severe endometriosis, 38% had hyperprolactinaemia. Hyperprolactinaemia was present in 10/37 (27%) infertile patients with endometriosis. Moreover, the PRL response to the LHRH/TRH test was exaggerated in patients with endometriosis compared to the normal controls. The PRL response was reduced after the treatment, but in patients with persistent endometriosis after danazol treatment, the PRL response was higher. In patients with exaggerated PRL response to LHRH/TRH tests before the treatment of endometriosis, danazol treatment was less effective [7].

The correlation of the prolactin serum levels and severity of endometriosis is a clinically important question. The study by Gregoriu et al. included 40 endometriosis patients with all stages of endometriosis. The basal and TRH stimulated prolactin serum levels were higher than normal in stage IV of endometriosis, but the mean basal serum prolactin concentrations were 12.5, 16.5, 19.5, and 26.5 ng/mL, and those after thyrotropin-releasing hormone (TRH) administration were 88.3, 114.2, 125.3, and 138.8 ng/mL in patients with stages I, II, III, and IV endometriosis, respectively. Patients were divided into two groups: 20 patients received GnRH analogue therapy for 24 weeks and another 20 patients did not receive any therapy for endometriosis. However, the pregnancy rate was not different between the two groups, but the patients who did not get pregnant had higher serum prolactin concentrations after TRH administration compared to those who conceived [12].

The authors of the study that included 20 patients with minimal and mild endometriosis, 7 fertile women with endometriosis, and 14 fertile women without endometriosis reported that prolactin serum levels higher than 20 ng/mL were observed only among infertile patients with stage I or II endometriosis (6/20). The serum oestradiol levels were lower in infertile patients with endometriosis than in fertile patients without endometriosis. Altered prolactin secretion in infertile patients with endometriosis was accompanied by luteal insufficiency, which was more frequent in infertile patients with endometriosis (78.9%) than in fertile patients with endometriosis (42.9%) or fertile women without endometriosis (0%) [13].

Another case–control study on this issue included 64 patients: 21 infertile patients with minimal/mild endometriosis, 10 fertile patients with minimal/mild endometriosis, and 33 fertile women without endometriosis. The results were similar to previous studies: the basal prolactin serum levels as well as prolactin response to TRH stimulation were higher in patients with endometriosis compared to the fertile controls. The results of this study also confirmed that infertile patients with endometriosis had lower serum oestradiol levels (48.40 pg/mL) than fertile patients with endometriosis (122.0 pg/mL) or fertile patients without endometriosis (52.10 pg/mL). The authors of the study concluded that decreased serum oestradiol levels and altered PRL secretion after TRH administration in infertile patients with minimal/mild endometriosis were related to ovulatory dysfunction and infertility in this group of patients without tubal occlusion [14].

A similar situation was observed in patients with advanced stages of endometriosis. In their study, Lima et al. included 18 infertile women with endometriosis stage I/II, 10 infertile women with stage III/IV, and 21 fertile women without endometriosis undergoing laparoscopy for tubal sterilisation and reported that the basal prolactin serum levels in patients with endometriosis stage III–IV and in patients with endometriosis stage I–II were higher (28.9 ± 2.1 ng/mL and 23.4 ± 3.7 ng/mL) when compared to the basal prolactin serum levels in fertile patients without endometriosis (13.2 ± 2.1 ng/mL). There were no differences between groups in prolactin concentration in the peritoneal fluid and in the follicular fluid. The authors concluded that high prolactin serum levels contributed to the subfertility in endometriotic patients and hypothesised that stress, which increases the prolactin levels, might be involved in the progression of disease and the development of endometriosis-induced infertility [15,45].

The authors of another cross-sectional study included 256 patients with infertility, and in 76/256 (29%), diagnostic laparoscopy revealed endometriosis. Prolactin serum levels were analysed in 76 infertile patients with endometriosis and 79 infertile patients without endometriosis, and confirmed higher prolactin levels in infertile patients with endometriosis (23.02 ± 1.25 ng/mL vs. 17.21 ± 1.22 ng/mL, respectively) and the correlation of prolactin serum levels with the stage of endometriosis (16.98 ± 1.29 ng/mL for stages I; 18.07 ± 1.50 ng/mL for stages II; 25.59 ± 1.96 ng/mL stages III–IV). Hyperprolactinaemia was registered only in patients with higher stages of endometriosis [16].

The recent study conducted by Pedachenko et al. (2021), which included 72 infertile women with endometriosis and 77 infertile women without endometriosis, also confirmed that the baseline prolactin concentrations were significantly higher in infertile women with endometriosis (16.9 ± 5.7 ng/mL) compared to the prolactin levels in infertile women without endometriosis (15 ± 4.3 ng/mL; *p* = 0.023) [18].

There is also a question of whether prolactin serum levels are valid as diagnostic criterium for the presence of endometriosis as well as whether there are differences in the prolactin serum levels between stages of the disease. Mirabi et al. (2019) reported in their retrospective cohort study, which included 114 infertile women with endometriosis and 101 infertile women without endometriosis, that the prolactin serum levels were higher in infertile patients with endometriosis compared to infertile patients without endometriosis: 23.42 ± 34.05 ng/mL in the infertile women with endometriosis stages I and II; 31.62 ± 38.09 ng/mL in the infertile women with endometriosis stages III and IV; and 17.88 ± 12.81 ng/mL in the group of infertile women without endometriosis. Significant differences in the prolactin serum levels were only registered between the group of infertile patients with endometriosis stages III/IV compared to infertile patients without endometriosis. PRL values with a cutoff set at 20.08 ng/mL had a sensitivity of 0.74 and specificity of 0.54 in detecting disease stages III/IV vs. I/II. PRL values with a cutoff set at 17.5 ng/mL, which had a sensitivity of 0.64 and specificity of 0.63 in segregating subjects with and without endometriosis [17].

Another study reported that prolactin serum levels could be used as a biomarker for peritoneal endometriosis in combination with the CA-125 levels. The study, which was performed with 97 participants, 63 women with peritoneal endometriosis and 34 healthy women, revealed that the serum CA-125 and prolactin levels assessed together, and considering the cutoffs for CA-125 (19.9 U/I) and prolactin (14.8 ng/mL), had acceptable sensitivity and specificity (77 and 88%, respectively) and a high negative predictive value (97%) to diagnose peritoneal endometriosis [59]. The authors of a recent study proposed a panel of inflammatory markers, particularly IL-6, PRL, and CA 125, as a useful tool to identify women with advanced endometriosis who could qualify for treatment [60]. However, a meta-analysis on 122 blood markers for the non-invasive diagnosis of endometriosis including the prolactin serum levels stated that despite the high specificity, the sensitivity for the prolactin serum levels was low and the thresholds remained unacceptably low, even for a triage test, and the data regarding prolactin and other hormonal biomarkers such as oestradiol, progesterone, LH, and FSH for the diagnosing of endometriosis were not sufficient to draw meaningful conclusions [61]. Moreover, the authors concluded that none of the blood biomarkers for the non-invasive diagnosis of endometriosis displayed enough accuracy to be used clinically outside a research setting [61].

Similarly, the authors of a recent meta-analysis comparing the diagnostic accuracy of different hormonal biomarkers for diagnosing endometriosis reported low sensitivity (0.45) but high specificity (0.92) for serum prolactin when compared with other hormonal markers such as aromatase, human chorionic gonadotropin/luteinising hormone receptor, oestrogen receptor (ER)-α, ER-β, oestrogen sulfotransferase, and 17β-hydroxysteroid dehydrogenase type 2. The authors concluded that because of the moderate quality of the included studies and the limited sample size, their results required more research to validate [62].

The problem of possible connections between hyperprolactinaemia and endometriosis-related infertility is further complicated with the timing of the sampling, which is particularly important for prolactin, as prolactin serum levels have a specific diurnal pattern. An excellent study including 55 women with endometriosis-related infertility was performed more than 30 years ago and showed that nocturnal prolactin secretion may be altered in infertile women with endometriosis, with an exaggerated and prolonged nocturnal peak. The only objection might be that the blood samples were collected throughout the night preceding surgery, considering the impact of stress on prolactin secretion. The authors concluded that “alteration in PRL dynamics may contribute to infertility in women with endometriosis and may be a part of the pathophysiology of this disease’’ [6].

In contrast to the results of the above-mentioned studies on the prolactin serum levels in patients with endometriosis-related infertility, there have been studies denying any relationship of prolactin and hyperprolactinaemia in the development of endometriosis. One of the first studies denying the involvement of hyperprolactinaemia in endometriosis-related infertility reported no differences in the mean prolactin serum level between 43 infertile patients with endometriosis (372 mIU/L, range 187–752 mIU/L) and 36 infertile patients with normal pelvic findings (333 mIU/L, range 124–767 mIU/L), both being in the normal range for the population (less than 540 mIU/L). The author concluded that there was no evidence to implicate raised prolactin levels as a cause for infertility in patients with endometriosis [8]. The authors of another study that involved 70 patients with endometriosis did not find any significant relationships between the basal prolactin serum levels and stage of endometriosis or the outcome of the infertility treatment, but even this study reported a significantly greater prolactin response after 500 micrograms of TRH injection in the patients who did not become pregnant than those who did [11].

The results of the studies on the effects of the treatment for endometriosis on the prolactin serum levels are also contradictory. One study investigating the effect of 6 months of danazol treatment found the absence of a significant difference in the basal prolactin levels as well as in the response to the TRH and insulin challenge tests between the controls and patients with endometriosis, before and after danazol treatment. The authors concluded that hyperprolactinaemia should not be considered as a cause of infertility in endometriotic patients, hypothesising that the significant reduction in prolactin response to challenging tests during danazol treatment may be associated with low oestrogen levels caused by medication [9].

The authors of another study on the effects of danazol treatment for endometriosis also reported the absence of a significant difference in the basal PRL levels as well as its response to the TRH in patients with endometriosis before and after danazol treatment and concluded that hyperprolactinaemia should not be considered as a cause of infertility in endometriotic women [10].

Nowadays, GnRH analogue therapy is standard for the treatment for endometriosis. In a prospective pilot study, Marschalek et al. reported that gonadotropin releasing hormone (GnRH) agonist therapy was capable of influencing the prolactin serum levels during therapy. The prolactin levels were unchanged after 4 weeks, but significantly decreased 8 and 12 weeks after the first leuporeline administration. The authors suggested that the decrease in prolactin levels might contribute to the known effect of GnRH treatment in endometriotic patients via the suppression of vascular endothelial growth factor expression in endometriotic lesions [63].

Some studies have suggested the possible beneficial effects of dopamine agonists in the treatment of endometriosis and adenomyosis. A clinical study by Gómez et al. reported beneficial effects of systemic hyperprolactinaemia treatment on peritoneal endometriosis: oral quinagolide 75 μg/d for 18–20 weeks reduced peritoneal endometriotic lesions where there was a 69.5% reduction in the size of the lesions and complete vanishing of the lesion in 35%. Immunohistochemical evaluation revealed interference with angiogenesis, enhanced fibrinolysis, reduced inflammation, and the downregulation of VEGF/VEGFR2, three proangiogenic cytokines (CCL2, RUNX1, and AGGF1), and plasminogen activator inhibitor (PAI) 1, a potent inhibitor of fibrinolysis, in the specimens of residual lesions that were surgically excised during the second look laparoscopy after the treatment. The results are promising, but a limitation of this study was the small number of patients with hyperprolactinemia-associated endometriosis (only nine patients were included) [64]. Vaginal bromocriptine (at a dose of 5 mg daily) significantly reduces heavy menstrual bleeding and pain in women with diffuse adenomyosis [65,66]. A recent study [67] reported that vaginal bromocriptine treatment (5 mg for 6 months) did not change the prolactin mRNA expression and concentration of prolactin in endometrial tissues, but inhibited the proliferation of endometrial tissue in adenomyosis as well as the capability of adenomyotic tissue to migrate in part through the regulation of dysregulated micro RNAs and signalling pathways associated with the proliferation of endometrial stromal cells.

## 8. Discussion

It is well-known that infertile patients with endometriosis have an already reduced ovarian reserve, impaired follicular development, and poor oocyte and embryo quality [68,69], and these findings could be associated with ovulatory disfunction caused by the prolactin hypersecretory state. Therefore, according to the cited clinical and experimental studies, explanations for infertility in patients with associated prolactin hypersecretory state and endometriosis without tubal obstruction, besides central defects in prolactin secretion, include impaired follicular development and ovulatory dysfunction with poor oocyte and embryo quality and more severe luteal phase defects. The results of most studies (Table 1) on basal and stimulated prolactin serum levels suggest that some infertile women with endometriosis exhibit a greater capacity for basal and stimulated prolactin secretion than normal fertile women, where the consequence is more severe luteal phase defects as a result of the association of hyperprolactinaemia and endometriosis. However, the results of the studies on this issue are not uniform, as other authors have reported quite contradictory results [8,9,10,11] denying the role of prolactin in endometriosis without tubal occlusion as a factor for the development of infertility and have focused on impaired ovarian reserve and reduced ovarian response in endometriosis, as indicated by elevated FSH and lower anti-Mullerian hormone. Such studies did not detect an increase in the prolactin levels in infertile women with endometriosis and ruled out hyperprolactinaemia as a cause of infertility in such patients.

A possible explanation of such different results could be the complexity of influences involved in the etiopathogenesis of hyperprolactinaemia and endometriosis. Stress, high levels of anxiety, depression, and other psychiatric disorders have been reported in infertile women with endometriosis as well as the impact on prolactin secretion of the medications used for the treatment of such disorders [70,71].

Sleep disturbances and stress could also be associated with higher serum prolactin levels, contributing as additional etiological factor for infertility [72]. This is in agreement with the conclusions of a previously mentioned study [6] that demonstrated that the alterations in prolactin circadian rhythm with exaggerated and prolonged nocturnal prolactin peak could also contribute to infertility in patients with endometriosis.

The essential question is what is the primary cause, and what is the consequence: hyperpolactinaemia that potentiates the development of endometriosis or enough endometriotic tissue that increases prolactin whether on a local or systemic level [73]. The parietal peritoneum is innervated largely by somatic nerves, and it has been suggested that endometriotic tissue could stimulate the peripheral nerves, leading to prolactin release, similar to the effect of suckling on prolactin secretion [5].

An important question for clinical practice is the possible effects of elevated prolactin serum levels on pelvic pain associated with endometriosis. Prolactin regulates the activity of nociceptors and causes hyperalgesia in pain conditions [74]. There is a possibility that increased prolactin serum levels in women with endometriosis could promote pelvic pain in these patients by dysregulating prolactin receptor expression. This is supported by the findings of preclinical studies that have demonstrated the capability of high prolactin serum levels or possibly repeated surges of prolactin to sensitise nociceptive neurons and promote pain in women [75]

Another clinically important question is the existence of some confounding factors that could indirectly affect the relationship between hyperprolactinemia and endometriosis-related infertility. Endometriosis itself is associated with autoimmune diseases such as systemic lupus, Sjögren’s syndrome, rheumatoid arthritis, autoimmune thyroid disorder, coeliac disease, multiple sclerosis, and inflammatory bowel disease [18,76]. Systemic hyperprolactinaemia is described in hepatic and renal failure, conditions that could complicate some of the above listed autoimmune diseases in which endometriosis is frequently present (e.g., systemic lupus, etc), representing a diagnostic and treatment confounding factor. This is also the truth with the presence of hyperprolactinaemia in patients with primary and subclinical hypothyroidism [77], which could be factors contributing to infertility in patients with and without endometriosis. This is also the case with stress, sleep disturbances [70,71], and some medications used to treat anxiety or depression frequently present in patients with endometriosis-related infertility, all of which are known to cause hyperprolactinaemia [72,78]. Interestingly, none of the cited clinical studies on the impact of prolactin/hyperprolactinaemia on endometriosis and endometriosis-related infertility have reported the mentioned factors as exclusion criteria.

On the other hand, the possible influence of prolactin on the development of infertility in endometriosis patients is not only the influence of systemic elevated prolactin levels or occult hyperprolactinaemia, but rather on the local level, as local prolactin acts as a cytokine, mitogen, and immunomodulator, and endometriotic tissue is susceptible to such actions. Elevated prolactin can also have modulatory effects on metabolic/endocrine and the immune systems. Endometriosis is a chronic inflammatory and hormone-dependent disease, and the immune system (systemically and locally in the endometrium, pelvic endometriotic lesions, and peritoneal fluid) is believed to play a central role in its aetiology, pathophysiology, and associated morbidities of pain, infertility, and poor pregnancy outcomes [55]. The action of local prolactin is related to the presence of prolactin receptors in endometriotic tissue. Again, the results of studies on this issue are not uniform. Some studies found a low expression of prolactin receptors in endometriotic tissue in the mid-follicular phase compared to the normal endometrium [44]. On the other hand, the authors of another study demonstrated that prolactin receptor blockade with antibodies completely inhibited endometriosis and concluded that enhanced local prolactin-mediated signalling in endometriotic lesions might contribute to the development of endometriosis [36].

The results of other experimental studies also support the role of local prolactin in the development of endometriosis [39,40]. These findings suggest that endometriotic cells have a different prolactin receptor gene expression pattern to normal endometrial cells, which could be a consequence of the fact that endometriotic cells are located outside the uterus, in a different environment with an altered concentration of hormones and other factors important for the expression of prolactin or steroid receptors.

The presence and amount of prolactin and steroid receptors in endometriotic tissue is not the only problem. The response to hormones in endometriotic tissue is also important. A normal menstrual cycle depends on tightly regulated oestrogen, progesterone, and other hormones and their signalling mechanisms. Every imbalance disrupts complex regulation, and with repeated altered cycles, these alterations accumulate, leading to irreversible or a hardly reversible and curable state. There is also the possibility of the existence of some unknown factor or disrupted signalling pathway that could interfere with pituitary prolactin secretion or local prolactin action, which is at the same time responsible for subfertility or infertility in some patients with endometriosis.

According to the reported studies, it seems that local prolactin, acting as an autocrine or paracrine factor, is more important in the development of endometriosis and adenomyosis than the prolactin serum levels. Further studies are needed to clarify the exact role of elevated PRL and PRL receptors as well as the associated mechanism in the pathogenesis of endometriosis and adenomyosis.

The effect of the treatment for hyperprolactinaemia and the effect of prolactin lowering medication on endometriosis are additional problems. Again, the results of the reported studies are contradictory. It seems that danazol treatment of endometriosis does not lower the basal prolactin level as well as the response to the TRH and insulin challenge tests [9,10].

The results of the clinical studies on the impact of prolactin lowering medication suggest the possible beneficial effects of dopamine agonists in endometriosis/adenomyosis [52,64,65,66,67].

The above-mentioned would be an explanation for the use of prolactin lowering medication in infertile patients with endometriosis and hyperprolactinaemia, but well-controlled clinical studies including an adequate number of infertile patients with an association of hyperprolactinaemia and endometriosis are still missing. Experimental studies support these clinical findings. Quinagolide was capable of reducing endometriotic lesions with a significant regression in endometriotic implants and also significantly reduced the peritoneal fluid levels of IL-6 and VEGF in rats transplanted with their own uterine fragments [79]. Another experimental study on mice demonstrated that prolactin-lowering drugs such as dopamine 2 receptor agonists effectively reduced endometriotic lesions in mice through the modulation of angiogenesis [80].

However, dopamine agonist treatment for endometriosis/adenomyosis is not routine, and further studies are needed to clarify the use of dopamine agonists in the treatment of endometriosis-induced infertility [81]. On the other hand, it is known that dopamine 2 receptor agonists such as bromocriptine and quinagolide are not able to block extrapituitary prolactin production due to different promotor usage for pituitary and extrapituitary prolactin synthesis in primates, at least in human uterine decidualised endometrial cells and lymphocytes [82].

Treatment options that are capable of influencing the local prolactin levels seem to be more promising and involve the treatment of endometriotic patients not only with prolactin-lowering drugs, but also prolactin monoclonal antibodies, prolactin receptor antibodies, and prolactin receptor antagonists. It was proposed that targeting prolactin and prolactin receptors may improve the management of the pain associated with endometriosis [83].

Humanised prolactin neutralising monoclonal antibodies have been discovered and characterised. It has been demonstrated that these antibodies produce concentration-dependent and complete inhibition of prolactin signalling at the hPRL receptor [84]. Clinical studies on the use of PRL neutralising monoclonal antibodies are still missing. According to the results of the previously mentioned experimental study that investigated the effects of prolactin receptor blockade in a murine endometriosis interna model [36], it seems that prolactin receptor antibodies may present a novel and efficient treatment option for endometriosis. However, there have been no reported clinical studies to test this hypothesis.

Prolactin receptor antagonists preventing endogenous prolactin from exerting its action via competitive mechanism for receptor binding have been proposed as a novel treatment option. Prolactin-receptor antagonists are engineered prolactin mutants capable of binding but not activating the prolactin receptor, thus preventing the actions of endogenous prolactin by competing for receptor binding. A new generation of human prolactin mutants has recently developed been that have no residual agonistic activity, behaving as pure antagonists [85]. It seems that this approach would be more efficient than dopamine agonists, capable of controlling both pituitary and extrapituitary prolactin activity. Adequate clinical studies are still missing.

The lack of adequate clinical studies regarding the beneficial effects of the treatment for hyperprolactinaemia on endometriosis-related infertility is the main problem. It is also well-known that the population of endometriotic patients is sufficiently heterogenous, making it almost impossible, despite every effort, to form a homogenous group of patients, especially when infertile patients are studied. There is also the problem of being able to recruit an appropriate number of patients, so only multicentric studies can provide the answer to this question.

The strengths of this review are as follows: a large number of references, covering long period of time, a thorough search of the current literature including studies written in languages other than English, data extraction by two independent reviewers, and the inclusion of studies that reported negative findings (i.e., studies denying a connection between prolactin/hyperprolactinaemia and endometriosis).

The main limitation of this review is that there was a low number of small, heterogeneous clinical studies on the connection of prolactin/hyperprolactinaemia and endometriosis-related infertility, especially on the outcomes of infertility treatment in patients with associated hyperprolactinaemia and endometriosis. There have been no well-controlled multicentric clinical studies on this issue. Furthermore, with the exception of one study performed 30 years ago, there have been no studies that have included circadian prolactin variation (i.e., nocturnal peak) in patients with endometriosis-related infertility.

## 9. Conclusions

Endometriosis and hyperprolactinaemia are conditions that might lead to infertility as a consequence, moreover, the association of endometriosis-related infertility and hyperprolactinaemia could be very resistant to treatment. The existence of extrapituitary prolactin and its role as a cytokine at the local level as well as the fact that the endometrium is one of the sources of extrapituitary prolactin suggest a possible relationship between prolactin/hyperprolactinaemia and endometriosis-related infertility. There are experimental and clinical studies that support such a connection, but final proof is still missing. Further clinical studies with an adequate number of infertile patients with endometriosis related-infertility and hyperprolactinaemia are needed to resolve this issue and determine adequate treatment in such patients.

## Figures and Tables

**Table 1:** Clinical studies on the influence of prolactin and hyperprolactinaemia in endometriosis.

Study—Authors	Study Design	PRL Levels in Patients with Endometriosis
Muse (1982) [5]	Prospective cohort study *n = 27*	Basal PRL concentration ↑ PRL response to TRH ↑
Radwanska et al. (1987) [6]	Prospective cohort study *n = 55*	Prolactin nocturnal peak↑ in endometriosis
Acién et al. (1989) [7]	Prospective cohort study *n = 66*	Basal PRL levels ↑ PRL response to LHRH and TRH ↑
Arumugam (1991) [8]	Prospective cohort study *n = 79*	No differences in basal PRL
Panidis (1992) [9]	Prospective cohort study *n = 20*	No differences in basal and TRH/insulin stimulated PRL before and after danazol treatment
Matalliotakis (1996) [10]	Prospective cohort study *n = 20*	No differences in basal and TRH stimulated PRL levels before and after danazol treatment
Machida et al. (1997) [11]	Prospective cohort study *n = 70*	No differences in basal PRL, but PRL response to TRH in-creased in patients who did not get pregnant
Gregoriu et al. (1999) [12]	Prospective cohort study *n = 40*	Basal and TRH stimulated PRL levels ↑
Cunha Filho (2001) [13]	Case–control study *n = 41*	Basal PRL levels ↑
Cunha Filho (2002) [14]	Case–control study: *n = 64*	Basal PRL levels ↑ PRL response to TRH ↑
Lima (2006) [15]	Case–control study *n = 49*	PRL serum levels ↑ in endometriosis stages III/IV and in stages I/II
Esmaeilzadeh (2015) [16]	Cross-sectional study *n = 256*	PRL levels ↑
Mirabi (2019) [17]	Retrospective cohort study *n = 215*	PRL serum levels ↑ in patients with III/IV stages.
Pedachenko (2021) [18]	Cross-sectional study *n = 149*	Basal PRL ↑ in endometriosis

Explanations: PRL–prolactin, TRH—thyrotropin-releasing hormone, LHRH—luteinizing hormone-releasing hormone, TSH—thyroid-stimulating hormone, ↑—PRL serum levels increased.

## Data Availability

The data analysed during this study are included in the published article and the manuscript conforms with the MDPI editorial policies and ethical policies.
